# Tracking the conical intersection dynamics for the photoinduced Jahn–Teller switch of a Mn(iii) complex†

**DOI:** 10.1039/d4sc00145a

**Published:** 2024-06-27

**Authors:** Ryan Phelps, Eleftheria Agapaki, Euan K. Brechin, J. Olof Johansson

**Affiliations:** a EaStCHEM School of Chemistry, University of Edinburgh David Brewster Road EH9 3FJ Edinburgh UK olof.johansson@ed.ac.uk

## Abstract

Octahedral Mn(iii) ions predominantly exhibit an axially elongated Jahn–Teller (JT) distortion, which is responsible for their large uniaxial magnetic anisotropy. As a result, they are often used in the synthesis of single-molecule magnets (SMMs). Modulation of the JT distortion using femtosecond laser pulses could offer a route to controlling the magnetisation direction, and therefore is promising for the development of data storage devices that work on ultrafast timescales. Photoinduced switching of the distortion from an axially elongated to an axially compressed structure has been demonstrated for various Mn(iii) complexes. However, the dynamics around the region of the conical intersection for the photoinduced JT switch remains unclear. Here, ultrafast transient absorption spectra were recorded for solutions of tris(2,2,6,6-tetramethyl-3,5-heptanedionato)manganese(iii) (Mn(dpm)_3_) in ethanol to further explore the dynamics of the photoinduced JT switch. We observe the generation of a vibrational wavepacket on the excited state surface, which has a frequency of approximately 155 cm^−1^ and encompasses a JT-active vibrational mode. This coherent motion is maintained after passage through the conical intersection back to the ground state, which launches wavepackets along the ground state potential energy surface (PES) with frequencies of approximately 180 and 110 cm^−1^ that we assign to the elongated and compressed state, respectively. Inspection of the relative phases of the frequencies reveals phase shifts that are consistent with a one-mode reaction coordinate, and passes through the conical intersection at 1/4 and 3/4 of the excited state vibrational period. Our results provide direct insights into the non-adiabatic dynamics of Mn(iii) complexes, which can be used to guide the synthesis of optically controlled SMMs.

## Abstract 2

We show that the relative phases of vibrational coherences can be used to observe the motion and timescales towards a conical intersection in the photoinduced Jahn–Teller switch of a Mn(iii) complex using ultrafast transient absorption spectroscopy.

## Graphical abstract

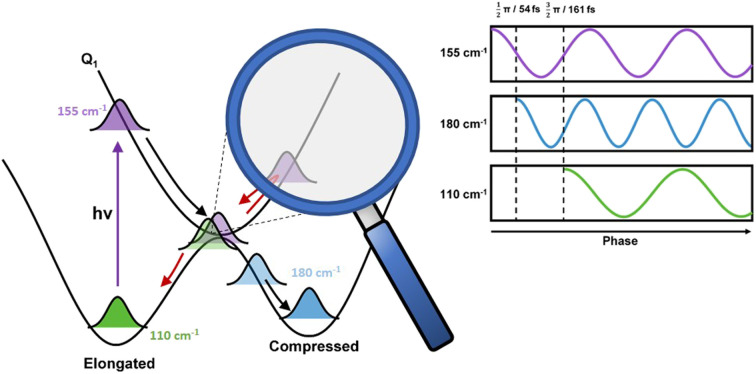

## 1 Introduction

**Fig. 1:**
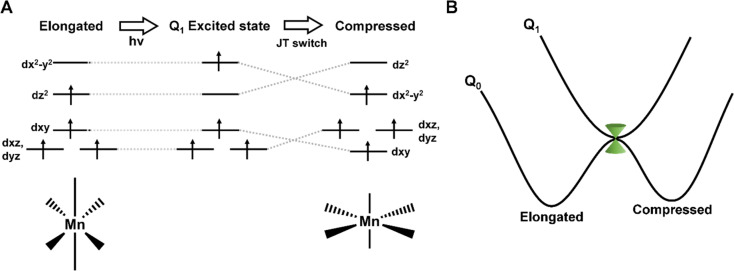
(A) Photoinduced Jahn–Teller switch of Mn(iii) complexes. (B) Schematic representation of the potential energy curves showing the states involved in the photoinduced JT switch. The position of the conical intersection is shown in green.

The field of molecular magnetism is rapidly evolving, aiming to manipulate magnetic properties at the molecular level.^1^ Being able to alter the magnetisation direction through interaction with femtosecond light pulses could offer a route to operate storage devices on unmatched timescales.^2^ Octahedral Mn(iii) ions predominantly exhibit an axially elongated Jahn–Teller (JT) distortion, typically resulting in a large uniaxial magnetic anisotropy. For this reason, Mn(iii) complexes have attracted attention as single-molecule magnets (SMMs).^3^ Previous work has shown that pressure can be used to switch the JT distortion, and alter the direction and magnitude of the magnetic anisotropy.^4^ This could be a way to overwrite stored information in an SMM from ‘zero’ to ‘one’. We have also shown that femtosecond pulses can be used to promote an electron from the d_*z*^2^_ to d_*x*^2^−*y*^2^_ orbital and cause a similar JT switch,^5–7^ as illustrated in Fig. 1. This shift in electron density causes a shortening of the axial bonds and elongation of the equatorial bonds, which is expected to modulate the amplitude and direction of the magnetic anisotropy. Consequently, the rapid motion out of the Franck-Condon region launches a vibrational wavepacket on the excited state potential energy surface (PES), identifying motions along the Q_1_ PES and the coordinates involved in the photoinduced JT switch. For Mn(acac)_3_ (acacH = acetylacetone), we found a long-lived signal (over several nanoseconds), which we attributed to the relaxation to the compressed ground state.^5^ This pathway was found to be switched off in complexes with more-restrictive ligands, suggesting dynamic control to the JT switch^5,7^

It has become well-established that conical intersections play a major role in photochemistry.^8–10^ However, our understanding of the dynamics around these regions of PES degeneracy remains incomplete. The Landau–Zener model has been successfully applied to many small molecular systems, where directions and velocities of approach to a conical intersection are crucial parameters.^11–15^ However, the highly multidimensional nature of PES means the one-mode Landau–Zener model often fails to fully describe the dynamics of the reaction.^10,16,17^ Instead, a full consideration of the intersection space must be considered.

Vibrational wavepackets evolve along a PES with a highly localised distribution of kinetic energies, which has been found to be responsible for some photochemical outcomes.^18–24^ In such cases, coherent motion can often be maintained in the photoproduct, due to the exceptionally fast passage between two PESs. This information provides valuable insights into the dynamics around the region of the conical intersection, and the reaction coordinates involved in photochemical transformations. Using this information, synthetic strategies have been devised to steer the dynamics around this region, and direct trajectories towards desired outcomes.^24^ Currently, our understanding of the photoinduced JT switch has focused on controlling reaction coordinates and the redistribution of localised vibrational energy.^5–7^ However, our knowledge of the dynamics around the region of the conical intersection and the factors that control the non-adiabatic transition remains elusive. To advance the field of ultrafast dynamics in SMMs, it is important to develop an understanding of the dynamics at the conical intersection, as this will determine the branching ratio of the axial and equatorial JT states, which in turn will determine the direction of the magnetisation. To enable photomagnetic recording of SMMs using fs laser pulses, this control will be imperative.

**Fig. 2:**
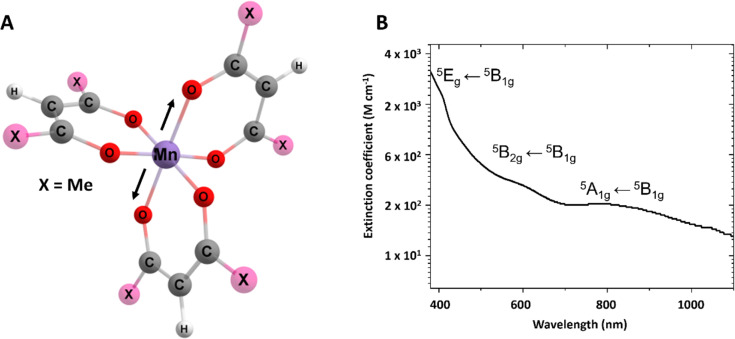
(A) Structure of Mn(dpm)_3_. Black arrows show the direction of the JT axis. (B) UV-Vis spectrum of Mn(dpm)_3_ in ethanol, the *Y* scale is logarithmic to more clearly see the three d–d transitions. Band assignments for the ^5^A_1g_ ← ^5^B_1g_(Q_1_), ^5^B_2g_ ← ^5^B_1g_(Q_2_), ^5^E_g_ ← ^5^B_1g_(Q_3_/Q_4_) are shown inset.

Here, we investigate the metal centred photoinduced dynamics of tris(2,2,6,6-tetramethyl-3,5-heptanedionato)manganese(iii) (shown in Fig. 2A and referred to herein as Mn(dpm)_3_) using transient absorption spectroscopy (TAS). The results show a photoinduced JT-switch and the formation of the compressed state. Careful inspection of the oscillations indicates a vibrational coherence is formed on the excited state surface, and this motion is maintained after passage through the conical intersection back to Q_0_. By inspection of the phases of the oscillations, we directly observe the motion through the conical intersection, and the timescale for the non-adiabatic transition after the initial launch of the excited state wavepacket. Being able to extract this information from the dynamics in other experiments, *i.e.* the crossing time and motions of the wavepackets, will allow us to understand which molecular properties are important to maximise the formation of the switched JT state, which is crucial for the control of magnetisation in SMMs.

## 2 Method

Mn(dpm)_3_ (Fig. 2A) was synthesised following a procedure previously reported,^25^ further details are outlined in the ESI.† The photoinduced dynamics initiated by the excitation of d–d transitions of Mn(dpm)_3_ (shown in Fig. 2B) were studied using transient absorption spectroscopy. The d–d transitions are assigned on the basis of the close correspondence to Mn(acac)_3_.^5^ Spectra were collected following the 640 nm excitation of 6.1 mM Mn(dpm)_3_ in ethanol (>99.8%, Sigma-Aldrich). Additional excitation wavelengths at 400 and 940 nm are shown in the ESI† to aid our interpretation. Although we are interested in the first excited quintet state, which can be directly accessed by pumping at 940 nm, the data are strongly affected by oscillatory signals that originate from the cuvette. Therefore, for this investigation we focus on the 640 nm excitation. Although we produce probe light down to 340 nm, the strong absorbance from the sample below 410 nm prevents acquiring reliable data within this region due to low probe intensities. Due to the stronger signals, we were able to perform experiments at 400 nm using a lower concentration, and therefore access probe wavelengths < 410 nm (Fig. S1 and S2†). However, in these experiments we expect participation from higher-lying states to participate in the dynamics. Raman spectra were measured using a Renishaw Raman microscope with a laser wavelength of 514 nm.

### 2.1 Transient absorption spectroscopy

The TAS instrumental setup has previously been described,^6,7^ and only essential experimental details are reported here. Samples were flowed at a rate of 16 μL min^−1^ through a Starna flow cuvette with a 1 mm pathlength and were intersected by pump pulses of 40 fs pulse duration. A pump wavelength of 640 nm and 9.4 mJ cm^−2^ fluence was used. The laser was set to a repetition rate of 1 kHz and intercepted by a mechanical chopper to produce pumped and unpumped spectra. The pump pulse was focussed on the sample using a spherical concave mirror to a beam radius of 100 μm at 1/*e*^2^ of the intensity. The excited samples were then probed by spatially overlapping and focusing a broadband probe generated in a 1028 nm pumped CaF_2_ plate. The probe has a radius of 50 μm at 1/*e*^2^ of the intensity at the sample position. The relative polarisation direction of the pump and probe was set to the magic angle (54.7°) and the intercept angle between the pump and probe was 1°. An optical delay line was used to control the relative delay of the pump and probe pulses to a maximum duration of ∼3 ns. Although we obtain a cross phase modulation artefact at time zero which remains for ∼180 fs, we are readily able to resolve frequencies shorter than 65 fs.^7^ It has been previously reported that the duration of the pump pulse is typically the limiting factor in the effective temporal duration for dispersed broadband detection schemes,^26^ and therefore we can expect our effective temporal resolution to be shorter than 40 fs.

After the sample, the probe was collimated and dispersed by a prism onto a fast charge-coupled device (CCD) camera (Entwicklungsbuero Stresing) equipped with a Hamamatsu S7031-0907 sensor with 512 × 58 active pixels. To reduce the shot-to-shot noise in the transient spectra, a portion of the probe beam was split before the sample and passed to a matched spectrometer.

### 2.2 Computational

Ground state geometry and vibrational frequency calculations have been conducted using the Gaussian 16 quantum chemistry package,^27^ using the PBE0 functional and the Def-2-SVP basis set. The computed Raman spectrum is in excellent agreement with the experiment, which we use to guide our assignment of coherences in our transient absorption data.

## 3 Results and discussion

**Fig. 3:**
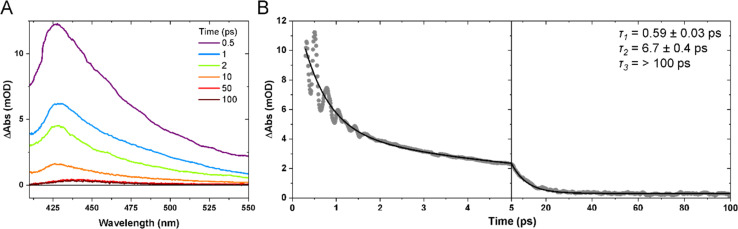
(A) TA spectra after 640 nm photoexcitation of 6.1 mM Mn(dpm)_3_ in ethanol at selected time delays. (B) Kinetic trace of the average intensity between 420–450 nm. Dots show experimental data points and the solid black curve is a kinetic fit to a biexponential function with a constant offset. Time constants (*τ*) extracted from the kinetic fits are shown inset.

The 640 nm photoexcitation of Mn(dpm)_3_ results in the TA spectra shown in Fig. 3A. A kinetic trace between 0–100 ps for the probe wavelength range of 420–450 nm is presented in Fig. 3B. An absorption band forms immediately within our instrument response function (<180 fs) and decays with time constants of 0.59 ± 0.03 and 6.7 ± 0.4 ps. We assign the absorption band to the first excited quintet (Q_1_) on the basis that we get the same spectral shape when we excite the lowest-lying d–d transition (d_*x*^2^−*y*^2^_ ← d_*z*^2^_) at 940 nm (Fig. S1 of the ESI†). More specifically we find no evidence of absorption bands at early time delays which would be indicative of higher lying states. An absorption signal remains for time durations greater than 100 ps, which we assign to the compressed ground state of Mn(dpm)_3_. We can discount vibrational relaxation as a cause of this shift which typically manifests itself as a blue shift since energy is lost to the surroundings. Our interpretation is consistent with our previous observations of Mn(acac)_3,_^5^ where we observe rapid population of the first excited quintet state and subsequent relaxation to the compressed state structure. For Mn(dmp)_3_ the compressed state spectrum is more red-shifted compared to the compressed state of Mn(acac)_3_, likely due to more significant changes in the crystal-field splitting as a result of the bulky tertiary-butyl substituents. The 0.59 and 6.7 ps time constants are likely convoluted with intramolecular vibrational energy redistribution and vibrational cooling dynamics. As we do not observe a ground state bleach signal, the signal decays to predominantly reform the ground elongated structure, and only a minor quantum yield of the compressed state.

### 3.1 Vibrational coherences

**Fig. 4:**
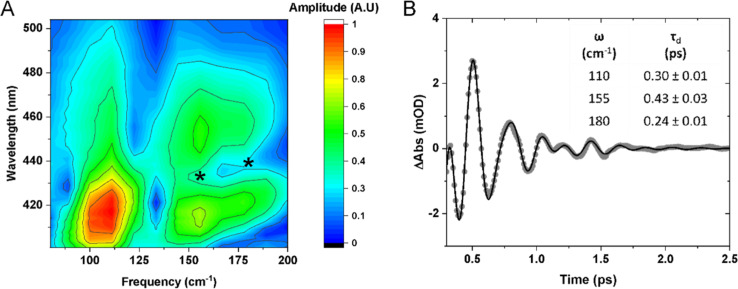
(A) FFT spectral map of the residuals extracted from the kinetic traces at each wavelength. * Show the position of the nodes. (B) Residuals from the kinetic fit shown in Fig. 3B. Dots show experimental data points and a solid black curve is fit with exponentially dampened cosine functions. The frequency (*ω*) of the cosine functions and their dephasing times (*τ*_d_) are shown inset.

Inspection of the signal intensity between 420–450 nm at early time delays (0–2.5 ps) shows oscillations, as shown in Fig. 3 and 4. We assign the oscillations to vibrational coherences caused by a switch from axial to equatorial JT distortion, similarly identified for Mn(acac)_3_.^5^ Fitting of the data to 3 exponentially dampened cosine functions reveals frequencies at 110, 155, and 180 cm^−1^, which agrees well with the Fourier transform of the data. The frequencies are shifted to lower frequencies with respect to those observed for Mn(acac)_3_ due to the bulky ^*t*^Bu-groups.^5^ The oscillations rapidly dephase with time constants of 0.30 ± 0.01, 0.43 ± 0.03, and 0.24 ± 0.01 ps, respectively. Compared to our previous observations with Mn(acac)_3_,^5^ we obtain an additional frequency component. We speculate that the lower frequencies observed in Mn(dpm)_3_ cause more well-defined and coherent motion.

#### 3.1.1 Assignment of vibrational coherences

**Fig. 5:**
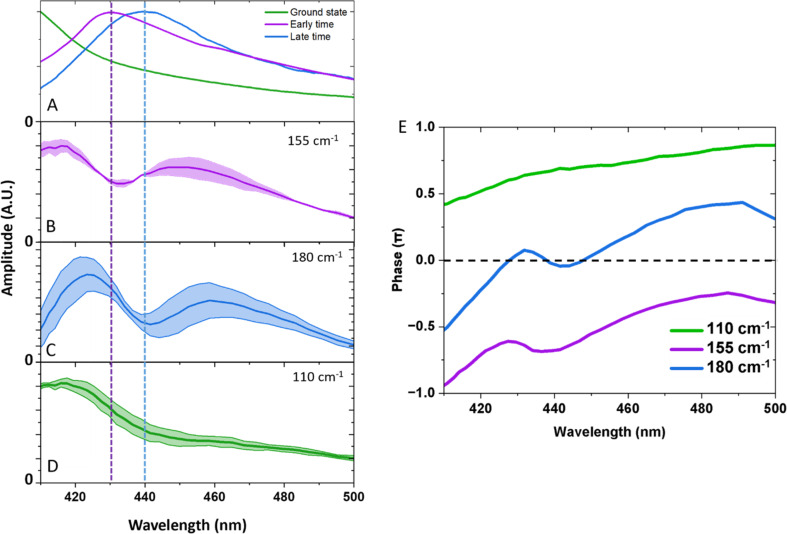
(A) UV-Vis spectrum of the ground state (green), early time spectra taken as an average from 0.3–0.5 ps (purple), and late time spectrum taken as an average between 30–100 ps (blue). Integrated frequency spectral amplitudes for: (B) 145–155 cm^−1^ (solid purple curve). (C) 180–190 cm^−1^ (solid blue curve) and (D) 100–110 cm^−1^ (solid green curve). Integration regions were selected to reduce the overlap of frequencies. Shaded regions show the extremes of these values. (E) Phases of the oscillations were extracted at the peak of the Fourier transform.

To identify which state the coherences originate from, we have inspected the phase information and compared the spectral amplitude of the coherences to the absorption profiles, as shown in Fig. 5A–D. Depending on the nature of the PES with respect to the probed state, the spectral amplitude can either (1) follow the amplitude of the absorption profile, caused by, for example, a modulation of the extinction coefficient, or (2) form an amplitude node with a π phase shift around the peak of the absorption band,^28,29^ a result of probing to a displaced PESs that causes a modulation of the band centre.^30,31^

Fig. 5A–D shows spectral slices of the 110, 155, and 180 cm^−1^ components. Amplitude nodes are evident for the 155 and 180 cm^−1^ oscillations around 430 and 440 nm respectively, indicating the probing to a displaced PES. This is further supported by the observed phase shifts across the probe spectral range as shown in Fig. 5E. The gradual change in phase as opposed to the expected abrupt shift can be explained due to anharmonic effects or closely spaced frequencies.^30^ The absorption profiles of the excited and compressed states were determined from the TA data set, extracting the average spectrum between 0.3–0.5 ps (predominantly excited state, and before a period of vibrational dephasing) and 30–90 ps (predominantly compressed state). The peak maxima are insensitive to the averaging window used (Fig. S3†). We find the amplitude node of the 155 and 180 cm^−1^ oscillations closely match the absorption peak of the early time and late time spectra, respectively. For this reason, we assign the 155 cm^−1^ wavepacket to the excited state and the 180 cm^−1^ component to the compressed state. Small discrepancies between the nodes and peaks could be a result of a variety of factors, such as: (1) deviations in the absorption maxima due to intramolecular vibrational energy redistribution and vibrational cooling dynamics. (2) Inhomogenous broadening, such that the complexes experience a different environment causing unique frequencies and absorption maxima.

From our previous arguments, a ground state coherence could expect to form an amplitude node at the peak of the UV spectrum, but this is outside of our probing window and is highly overlapped by intense bands. As we might expect oscillations to appear from these other bands, it is likely the amplitude spectra would be complicated as there would be frequency interference from other states. We discount assignment of the 110 cm^−1^ frequency to the excited and compressed states on the basis that we do not observe an amplitude node around the absorption maxima of either state, neither does the oscillation amplitude spectrum follow the shape of the absorption spectra. This makes a ground state coherence the most plausible assignment, which is further supported by the fact we see the oscillation amplitude is greatest along the red-wing of the absorption band, which is less spectrally congested. The maintenance of coherence after passage through the conical intersection unambiguously identifies an ultrafast non-adiabatic pathway to the compressed state, which occurs within a vibrational period (<215 fs). Because this timescale is convoluted with the cross-phase modulation artefact, we are unable to track the growth of the compressed state from a kinetic fit. Since we still observe the Q_1_ state in our measurements, a large portion of the wavepacket remains on the excited state potential energy surface. It is plausible that excess energy is redistributed into non-reactive modes which compete against this non-adiabatic pathway.

#### 3.1.2 Observation of the timescale through the conical intersection

**Fig. 6:**
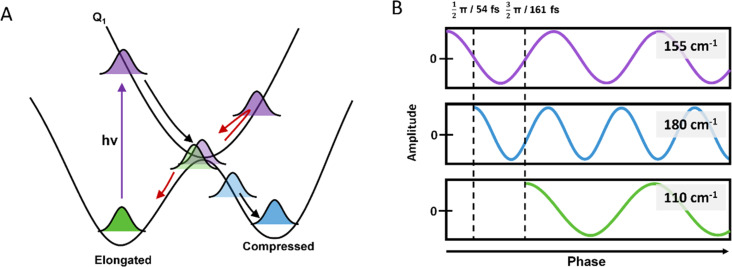
(A) Ground and excited state PESs of Mn(dpm)_3_. Photoexcitation of Mn(dpm)_3_ generates wavepackets on the excited (purple), compressed (blue) and elongated (green) states. The black arrows illustrate the formation of the compressed state upon the first pass of the conical intersection. Red arrows show the return of the wavepacket to form the elongated state. (B) Phase shifts of a cosine function generated immediately after photoexcitation (purple), at the first pass of the conical intersection (blue), and the second pass of the conical intersection during the return of the wavepacket (green).

To determine the timescale for the motion through the JT conical intersection more precisely, we monitor the phases of the wavepackets. Based on a one-mode reaction coordinate, the excited state wavepacket would be expected to reach the conical intersection every 1/4 and 3/4 of the vibrational period, as demonstrated in Fig. 6A. During the first pass, the approach direction should favour the compressed state, while at the second pass, the direction of the approach should favour the return to the elongated state. As a result, compressed and elongated state coherences would be launched when the phase of the excited state coherence reaches 1/2 π and 3/2 π respectively. Assuming the 155 cm^−1^ is the main reaction coordinate, this would correspond to launching of the wavepackets at 54 fs and 161 fs after the launch of the 155 cm^−1^ component, as shown in Fig. 6B. Herein, we will use units of fs to describe the phase shifts relative to the 155 cm^−1^ component for simplicity. We recognise exact phases can be difficult to extract due to the presence of the cross-phase modulation artefact around time zero. To extract phases we truncate our data sets and our analysis starts after 300 fs. Since these oscillations ring out to around 2 ps, we are able to accurately extract the frequencies and their relative phases. Since the relative phase are not altered by the finite temporal bandwidth of our pulse, and instead phase resolution is limited by the size of our FFT window we are able to determine relative phases with high accuracy. For example, with a ∼50 fs phase shift we would expect the 180 cm^−1^ component to be ∼200 fs out of phase with respect to the 155 cm^−1^ frequency within 4 oscillations, which we can readily resolve in our experiments.

**Fig. 7:**
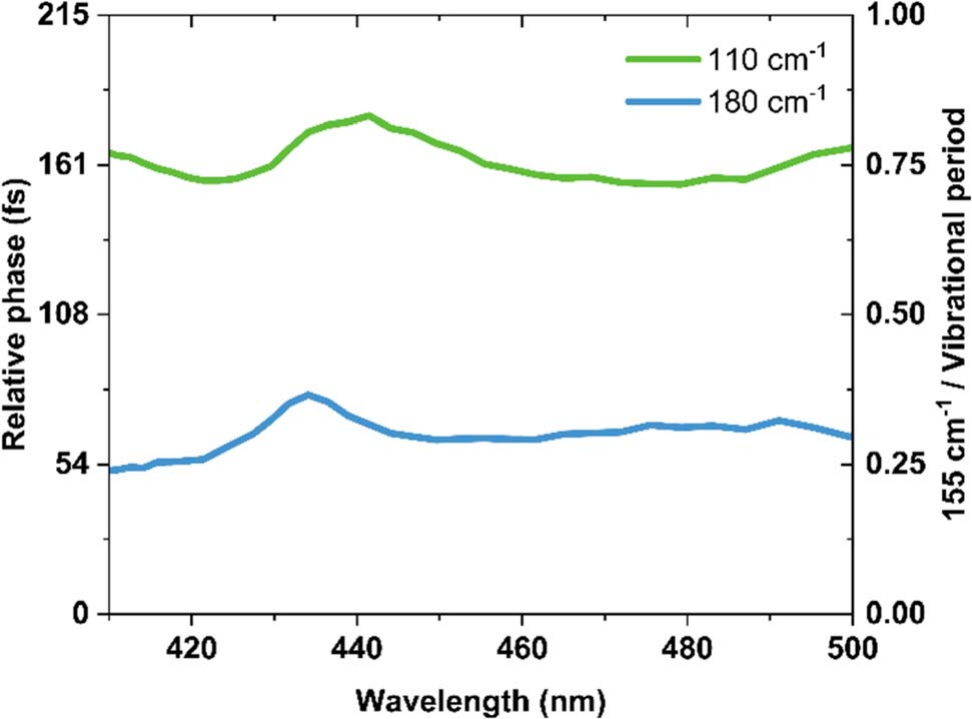
Phase shifts of vibrational coherences extracted from Fourier analysis relative to the 155 cm^−1^ component.

Fig. 7 shows the phase information extracted from the FFT analysis for the 180, 155 and 110 cm^−1^ oscillations. We found that relative to the launch of the 155 cm^−1^ wavepacket, the 180 cm^−1^ and 110 cm^−1^ components showed 64 ± 7 fs and 163 ± 7 fs phase shifts, respectively. The errors are determined from the standard deviation of the phase shifts across the probe spectral range, and do not encompass the finite bandwidth of our laser. These phase shifts are consistent with a one-mode reaction coordinate, and provide a direct method of monitoring the timescale for the passage through a conical intersection after the initial launch of the excited state wavepacket.

#### 3.1.3 – Reaction coordinate

**Fig. 8:**
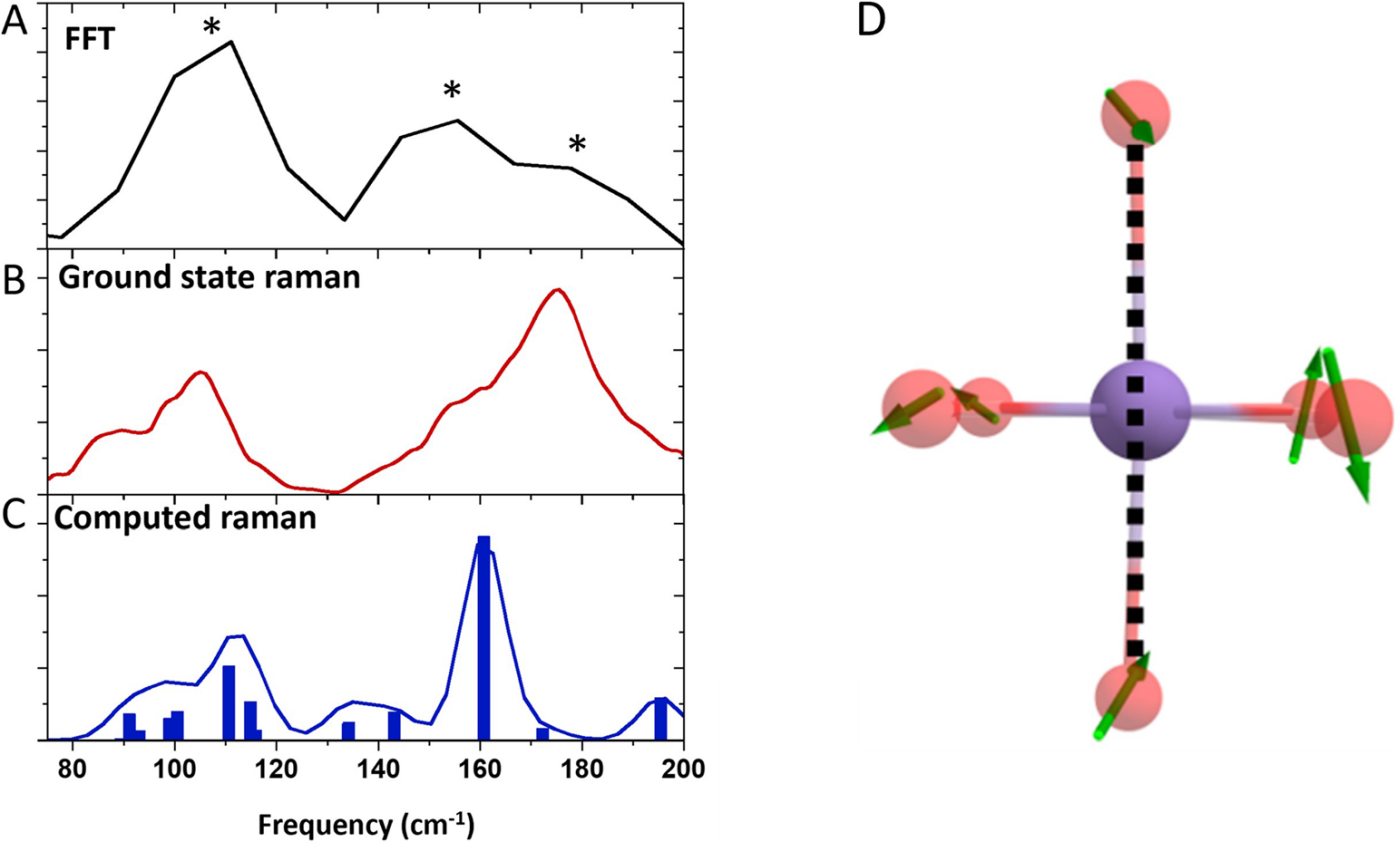
(A) FFT of the oscillations extracted from the kinetic traces. An average value across 400–500 nm was used. Asterix show the position of the 110 cm^−1^ (green) 155 cm^−1^ (purple) and 180 cm^−1^ (blue) components. (B) Ground state Raman of Mn(dpm)_3_. (C) Computed Raman of Mn(dpm)_3_. (D) Displacement vectors for the computed frequency at 110 cm^−1^ for the elongated state of Mn(dpm)_3_. The dotted line shows the JT axis. The hydrocarbon backbone has been removed for clarity.

To categorise the motions of the vibrational coherences, we compare the frequency assigned to the elongated state (110 cm^−1^) with vibrational frequency calculations. We obtain good agreement with the experimental Raman spectrum (Fig. 8A–C) and assign the observed 110 cm^−1^ frequency to *ω*_18_, shown in Fig. 8D and predicted around 110 cm^−1^. Because this coherence has been transferred from the excited state, we assume the other observed frequencies to have similar motions. The mode assigned collectively modulates the axial and equatorial bond lengths such that the compressed state can be accessed within a single reaction coordinate. Other frequencies around this wavenumber region also tend to be JT-active, but have motions which we do not expect to be involved, *i.e.*, asymmetric stretching of the axial bonds. It is worth mentioning that the excited and compressed state coherences match well with the ground state Raman spectrum, which could incorrectly identify the 155 and 180 cm^−1^ components as ground state coherences. The close correspondence to the ground state Raman spectrum can be understood by considering the motions of the nuclei. Since the motions we assign involve collective axial and equatorial stretching, a switch in the JT distortion will have little overall effect on the observed frequency as their bond length changes will effectively cancel their influence.

## 4 Conclusion

In conclusion, we have performed TAS measurements on Mn(dpm)_3_, which provide insights into the non-adiabatic dynamics of Mn(iii) complexes and their photoinduced JT switch. We have observed the generation of a vibrational wavepacket on the excited state surface that maintains coherent motion after passage through the conical intersection. The relative phase shifts of the frequencies are consistent with a one-mode reaction coordinate, which we assign to a JT-active vibrational mode. We demonstrate that we can directly track the motion through the conical intersection, and estimate the non-adiabatic transition to the ground compressed and elongated states to be launched at 1/4 and 3/4 of the excited state vibrational period, respectively. These findings could be used to guide the synthesis of optically controlled SMMs by applying this method for a variety of complexes, which will allow us to understand which molecular properties are important to maximise the formation of the switched JT state. Being able to optically switch the JT state of a SMM offers a promising route towards the development of data storage devices that work on ultrafast timescales. Further studies on the dynamics around the regions of PES degeneracy, such as conical intersections, are necessary to fully understand, predict, and control the outcomes of photochemical reactions in complex systems.

## Data availability

Data for this article, including transient absorption data for various wavelengths and Raman spectra, are available at https://doi.org/10.7488/ds/7761.

## Author contributions

R. P. performed the transient absorption experiments, computations, analysed the data, and wrote the manuscript. E. A. performed the synthesis, characterisation, wrote the synthetic methodology and reviewed the manuscript. E. K. B. and J. O. J. conceptualised the experiments, acquired funding, and edited the manuscript.

## Conflicts of interest

There are no conflicts to declare.

## Supplementary Material

SC-015-D4SC00145A-s001
