# Supplementary material for: Tracking the conical intersection dynamics for the photoinduced Jahn–Teller switch of a Mn(iii) complex
Source: Chem Sci. 2024 Jun 27;15(30):11956–64. doi: 10.1039/d4sc00145a (PMC11290422; doi:10.1039/d4sc00145a)
Supplement: SC-015-D4SC00145A-s001 [file SC-015-D4SC00145A-s001.pdf]

Electronic Supplementary Material (ESI) for Chemical Science.
This journal is © The Royal Society of Chemistry 2024

Supplementary Information (SI) for: Tracking the Conical Intersection Dynamics for the Photoinduced Jahn-Teller Switch of a Mn(III) Complex

Ryan Phelps, Eleftheria Agapaki, Euan K. Brechin, and J. Olof Johansson*
EaStCHEM School of Chemistry, University of Edinburgh, David Brewster Road, EH9 3FJ, Edinburgh, UK
*Email: olof.johansson@ed.ac.uk

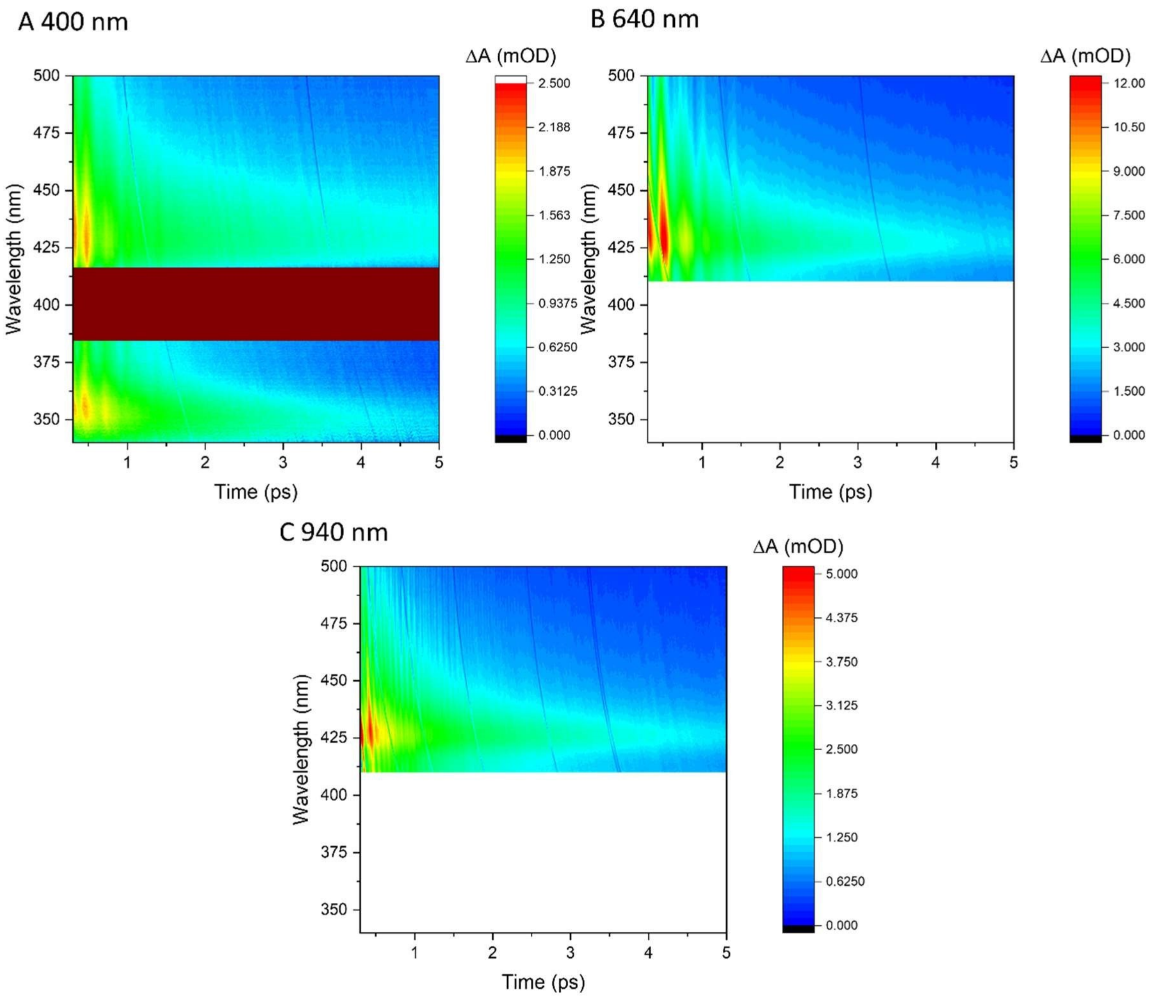

Figure S1 – TA spectra following the photoexcitation of $Mn(dmp)_3$ in ethanol at A – 400 nm, B – 640 nm, C – 940 nm. Due to the higher concentration used in the 640 and 940 nm data set, probe wavelengths were not accessible below 410 nm.

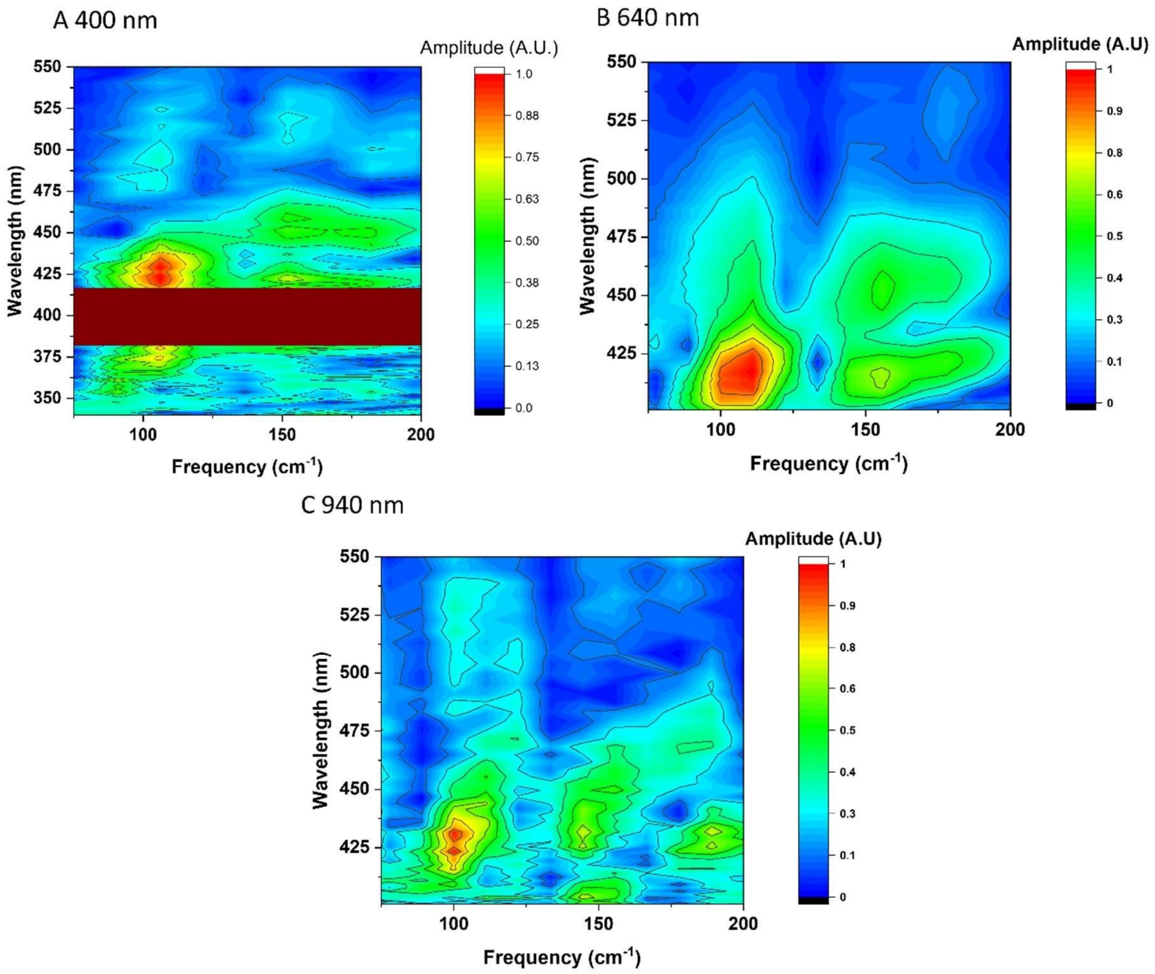

Figure S2 – FFT spectral map of the residuals extracted from the kinetic traces at each wavelength following the photoexcitation of $Mn(dmp)_3$ in ethanol at A – 400 nm, B – 640 nm, C – 940 nm. Due to the higher concentration used in the 640 and 940 nm data set, probe wavelengths were not accessible below 410 nm.

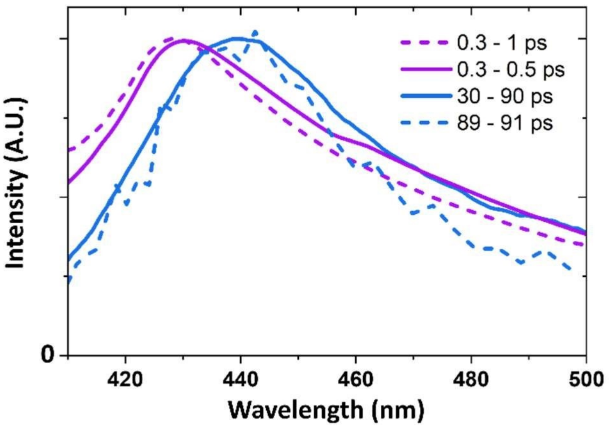

Figure S3 – Averaged TA spectra. Early time (purple) is shown between 0.3 – 0.5 (solid purple) and 0.3 – 1 ps (dashed purple). Late time is shown between 30 – 90 ps (solid blue) and 89 – 91 ps (dashed blue).

Synthesis

2,2,4,4-Tetramethylhept-3,5-dione (1.3 mL, 6.1 mmol, 3.0 equiv) was added to a solution of manganese(II)diacetate tetrahydrate (0.50 g, 2.0 mmol, 1.0 equiv) in methanol (4.5 mL) under air and the resulting yellow-green solution was stirred at 23 °C. A solution of sodium hydroxide (0.25 g, 6.1 mmol, 3.0 eq) in water (1.2 mL, deionized) was then added, whereas a green solid immediately precipitated. After diluting with methanol (5.5 mL), the reaction mixture was stirred at 23 °C under air for 13 h and filtered. The green-brown solid was dried in high vacuo at 60° C for 4 h and dissolved in hot isopropanol (15 mL). $Mn(dpm)_3$ precipitated partially upon cooling, whereas the precipitation was completed by adding water (3 mL) and the suspension was filtered. The resulting solid was suspended in pentane (10 mL) and the resulting suspension was filtered, removing some brown-red impurities. The pentane was removed under reduced pressure and the resulting olive green solid was dried under high vacuo for 12 h to give $Mn(dpm)_3$. Elemental analysis (%) calculated for $MnO_6C_{33}H_{57}$: C, 65.54 %; H,
9.50. Found: C, 65.59 %; H, 9.48 %.

Characterisation pXRD

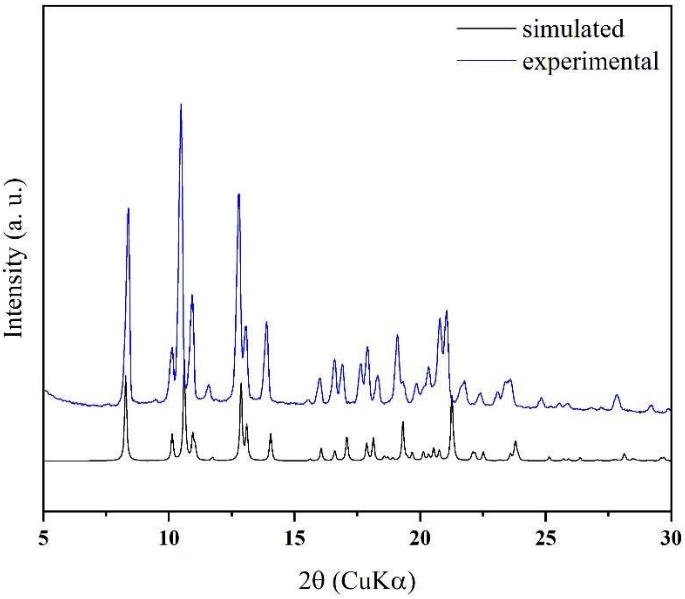

Figure S4 - Powder X-ray data for $Mn(dpm)_3$. The blue and black lines are the experimental and simulated data, respectively.

IR

The infrared spectrum contains all the characteristic vibrations (in cm^{-1}), which are in accordance with literature data [T. Nakamura, R. Tai, T. Nishimura and K. Tachibana, J. Electrochem. Soc., 2005, 152, C584]:

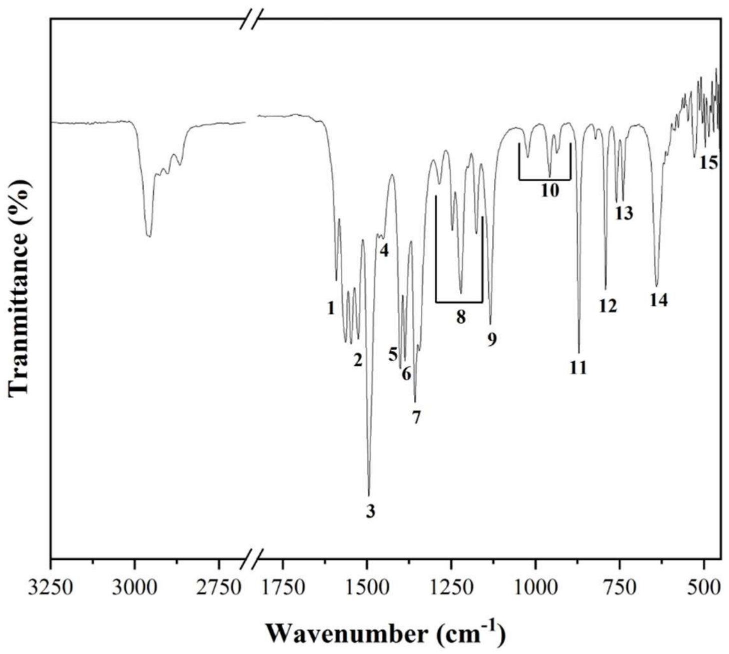

Figure S5 - IR spectra of $Mn(dpm)_3$ at RT.

Table S1 - Observed wavenumber assignments of the $Mn(dpm)_3$ complex.

Number	Wavenumber (cm^{-1})
1	1592, 1569
2	1548, 1528
3	1496
4	1452
5	1401
6	1387
7	1358
8	1285, 1246, 1223, 1175
9	1135
10	1024, 958, 935
11	871
12	792
13	759, 739
14	640
15	506, 479
